# Data documenting the performance of the PT/INR line correction method for reconciling INR discrepancies between central laboratory coagulation analyzers using different thromboplastins during the evaluation of a portable Coagulometer

**DOI:** 10.1016/j.dib.2017.11.020

**Published:** 2017-11-07

**Authors:** Wendy S. Baker, Kathleen J. Albright, Heidi Spratt, Megan Berman, Peggy A. Mann, Jaime Unabia, John R. Petersen

**Affiliations:** a University of Texas Medical Branch, Department of Preventive Medicine and Community Health, Galveston, TX 77555-1150, United States; b University of Texas Medical Branch, Department of Internal Medicine, Galveston, TX 77555-1167, United States; c University of Texas Medical Branch, Department of Clinics Administration & Support, League City, TX 77573-1210, United States; d University of Texas Medical Branch, Department of Pathology, Galveston, TX 77555-0609, United States

## Abstract

The data presented here was produced as part of an evaluation of the performance of the CoaguChek XS point-of-care coagulation analyzer, which is discussed in the research article “POCT PT INR – Is it adequate for Patient Care? A Comparison of the Roche Coaguchek XS vs. Stago Star vs. Siemens BCS in Patients Routinely Seen in an Anticoagulation Clinic” (Baker et al., in press) [1]. An effort to reconcile discrepancies in the patient INR result distributions from different central lab instruments (Stago Star and Siemens BCS) with the PT/INR line method is described (Poller et al., 2010, 2011; Ibrahim et al., 2011) [2], [3], [4]. While regression analysis of the ECAA Poller calibrant data provided a linear PT/INR line for all methods, Pearson's chi-squared and one-way repeated measures ANOVA analyses showed that central lab INR measurements continued to exhibit measurement site dependence after the PT/INR line correction was applied. According to paired t-test analysis, only the human thromboplastin dependent methods (CoaguChek XS and Siemens BCS both before and after PT/INR line correction) showed statistically significant agreement (*p*-value >0.05).

**Specifications Table**

**Table:** 

Subject area	Clinical chemistry, hematology
More specific subject area	Coagulation analysis, prothrombin time analysis, INR
Type of data	Figures (scatter plots, histograms), tables
How data was acquired	CoaguChek XS (Roche Diagnostics), Star Evolution (Stago), BCS XP (Siemens Medical Solutions) coagulation analyzers
Data format	Raw and analyzed
Experimental factors	Described in the methods section
Experimental features	Described in the methods section
Data source location	UTMB (Galveston, TX), ARUP (Salt Lake City, UT), CPL (Austin, TX)
Data accessibility	Raw sample data in Table 1 and ECAA standards data Ref. [1]

**Value of the data**

• The data indicate that although agreement between central lab methods was improved by the PT/INR line correction, the INR results continued to exhibit statistically significant site dependence.
• The raw data here may provide insight pertinent to INR standardization efforts.
• Although the PT/INR line correction method is designed to minimize INR variation due to differences in instrumentation, thromboplastin, and local ISI correction strategy, the data indicate a need for method innovation. Additionally, a method [5] which allows for standardization of INR results obtained from disparate sample types (e.g. whole blood and plasma) would be beneficial.

## 1 Data

**Fig. 1:**
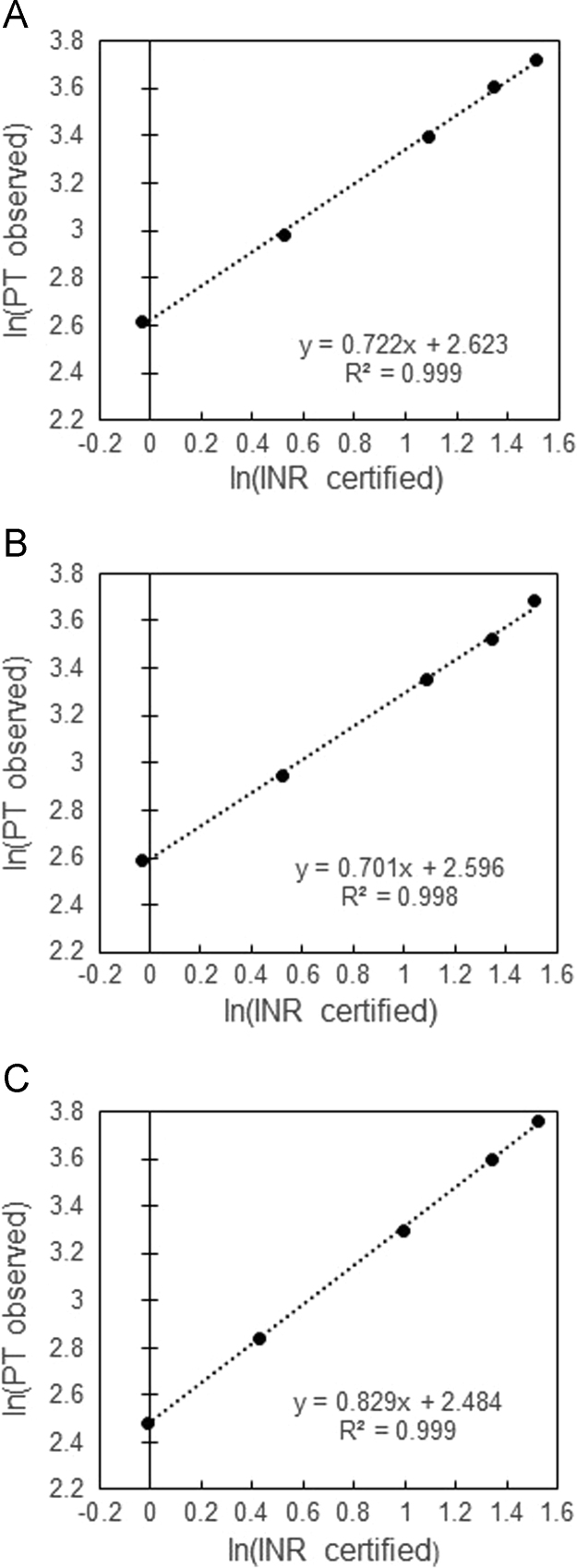
PT/INR calibration line and regression analysis generated from testing of ECAA Poller calibrants by (A) UTMB Stago Star Evolution, (B) ARUP Stago Star Evolution, and (C) CPL Siemens BCS XP.

Fig. 1 shows the European Concerted Action on Anticoagulation (ECAA) Poller calibrant-generated PT/INR lines for the Stago Star Evolution instruments at the University of Texas Medical Branch (UTMB, Fig. 1A), the Associated Regional and University Pathologists, Inc. (ARUP, Fig. 1B), and the Siemens BCS XP at Clinical Pathology Laboratories, Inc. (CPL, Fig. 1C). The calibrant data provide a linear fit to the model for all methods, as evidenced by their coefficients of determination (*R*^2^>0.998). The generation of the calibrations plots and PT/INR line method is described briefly in the methods section and detailed more fully by Poller et al. [2], [3], [4].

**Fig. 2:**
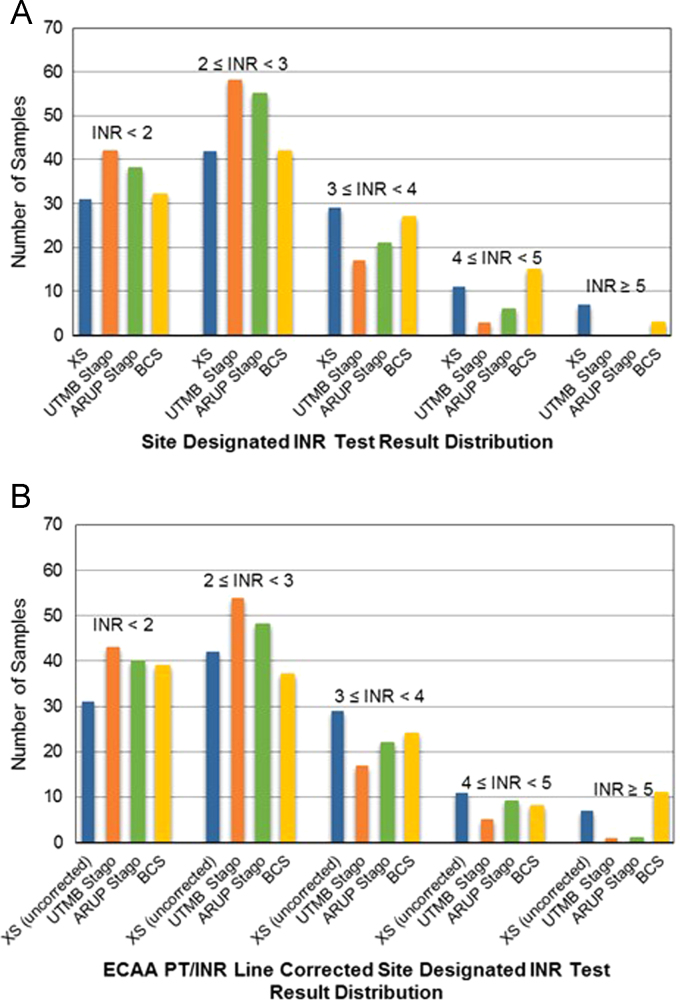
Sample population INR distribution (A) before (raw data) and (B) after PT/INR line correction (see Ref. [2]) using ECAA Poller standards.

The PT/INR line fit was employed to correct central lab INR results for 100 warfarin therapy and 20 control samples examined as part of a CoaguChek XS point-of-care device study, as detailed in Table 1 [1]. Fig. 2A shows the frequency distribution of the uncorrected INR values for the central lab and CoaguChek XS coagulometers. Visual inspection of the histograms in Fig. 2A show that the recombinant human thromboplastin based methods (Coaguchek XS and BCS) have similar INR frequency distributions. Additionally, the rabbit brain thromboplastin based methods (UTMB and ARUP Stago) INR frequency distributions exhibit a comparable pattern. In Fig. 2B the PT/INR line corrected INR results are shown for all central laboratory methods alongside the uncorrected CoaguChek XS data (the POCT device is only approved for analysis of whole blood samples thus could not be calibrated using the plasma-based ECAA Poller standards[5]). Visual inspection of Fig. 2B reveals no obvious improvement in the net agreement between methods, however, Pearson's chi-squared testing of the frequency distribution data shown in Table 2 provided *χ*^2^=33.36 with *p*=0.00005 (before PT/INR line correction) and *χ*^2^=25.56 with *p*=0.012 (after PT/INR line correction). The chi-squared test results showed that although the PT/INR line correction method failed to provide inter-method agreement at the *p*=0.05 threshold, improved agreement was observed.

**Table 1:** Raw and PT/INR line corrected sample data.

Sample	Roche XS		UTMB Stago			ARUP Stago			CPL BCS XP		
	Sec	INR	Sec	INR	PT/INR corrected	Sec	INR	PT/INR corrected	Sec	INR	PT/INR corrected
3N	11.40	1.00	12.60	0.90	0.83	12.00	0.90	0.87	9.90	0.90	0.80
10N	11.10	0.90	12.60	0.90	0.83	12.60	1.00	0.92	10.20	1.00	0.83
19N	11.60	1.00	12.50	0.90	0.82	13.00	1.00	0.96	11.30	1.10	0.92
20N	10.80	0.90	12.50	0.90	0.82	12.90	0.90	0.95	9.90	0.90	0.80
IN	11.70	1.00	13.00	1.00	0.87	12.80	1.00	0.94	11.10	1.10	0.90
2N	11.30	0.90	13.10	1.00	0.88	12.80	1.00	0.94	10.00	0.90	0.81
4N	12.20	1.00	13.30	1.00	0.90	13.50	1.10	1.01	10.60	1.00	0.86
5N	12.70	1.10	13.70	1.00	0.93	13.60	1.10	1.02	11.20	1.10	0.91
6N	11.00	1.00	13.40	1.00	0.90	13.10	1.00	0.97	10.30	1.00	0.83
7N	11.50	1.00	13.30	1.00	0.90	12.20	0.90	0.89	10.60	1.00	0.86
8N	11.40	0.90	13.10	1.00	0.88	13.10	1.00	0.97	10.30	1.00	0.83
9N	11.40	1.00	12.90	1.00	0.86	12.40	1.00	0.91	NA	NA	NA
11N	10.80	0.90	12.90	1.00	0.86	13.20	1.00	0.98	9.90	0.90	0.80
12N	11.30	0.90	13.30	1.00	0.90	13.00	1.00	0.96	10.40	1.00	0.84
13N	10.40	0.90	13.30	1.00	0.90	12.90	1.00	0.95	10.20	1.00	0.83
14N	10.60	0.90	13.50	1.00	0.91	13.30	1.00	0.99	10.80	1.00	0.87
15N	11.00	0.90	13.30	1.00	0.90	13.20	1.00	0.98	10.80	1.00	0.87
16N	11.30	0.90	13.00	1.00	0.87	12.90	1.00	0.95	11.10	1.10	0.90
17N	11.70	1.00	13.40	1.00	0.90	12.90	1.00	0.95	10.70	1.00	0.87
18N	11.30	0.90	13.00	1.00	0.87	13.00	1.00	0.96	10.60	1.00	0.86
10A	15.60	1.30	14.40	1.10	0.99	14.20	1.10	1.07	11.40	1.10	0.92
39A	14.20	1.20	15.60	1.20	1.11	15.10	1.20	1.16	11.80	1.10	0.96
1A	16.60	1.40	16.30	1.30	1.17	16.70	1.40	1.31	13.90	1.40	1.13
17A	16.20	1.40	17.30	1.40	1.27	16.50	1.40	1.29	14.50	1.40	1.18
44A	14.70	1.20	16.90	1.40	1.23	16.10	1.30	1.25	13.40	1.30	1.09
26A	21.80	1.80	19.10	1.60	1.45	19.50	1.70	1.59	19.90	2.00	1.62
34A	20.40	1.70	19.50	1.60	1.49	19.70	1.70	1.61	17.20	1.70	1.40
36A	22.70	1.90	19.60	1.60	1.50	19.30	1.70	1.57	19.60	2.00	1.59
6A	21.50	1.80	20.40	1.70	1.58	19.90	1.80	1.63	18.20	1.80	1.48
12A	24.10	2.00	20.10	1.70	1.55	19.20	1.70	1.56	16.90	1.70	1.37
20A	26.00	2.20	20.50	1.70	1.59	20.30	1.80	1.67	18.90	1.90	1.54
42A	23.80	2.00	20.00	1.70	1.54	20.30	1.80	1.67	18.80	1.90	1.53
14A	22.30	1.90	21.30	1.80	1.67	20.60	1.80	1.71	19.00	1.90	1.55
32A	23.30	1.90	21.20	1.80	1.66	21.00	1.90	1.75	19.90	2.00	1.62
47A	23.40	2.00	20.80	1.80	1.62	21.00	1.90	1.75	22.90	2.30	1.87
51A	25.20	2.10	20.90	1.80	1.63	20.30	1.80	1.67	20.60	2.10	1.68
77A	23.80	2.00	21.30	1.80	1.67	21.60	2.00	1.81	20.50	2.10	1.67
7A	27.00	2.20	21.60	1.90	1.70	21.80	2.00	1.83	23.60	2.40	1.92
24A	28.30	2.40	22.30	1.90	1.77	25.00	2.40	2.17	24.00	2.40	1.96
27A	24.80	2.10	21.80	1.90	1.72	21.40	1.90	1.79	19.30	1.90	1.57
35A	25.20	2.10	22.00	1.90	1.74	21.70	2.00	1.82	19.40	1.90	1.58
49A	26.00	2.20	21.80	1.90	1.72	21.50	1.90	1.80	21.50	2.20	1.75
18A	25.10	2.10	22.90	2.00	1.84	21.80	2.00	1.83	20.00	2.00	1.63
25A	26.00	2.20	23.00	2.00	1.85	22.90	2.10	1.95	19.60	2.00	1.59
73A	25.60	2.10	22.70	2.00	1.82	22.10	2.00	1.86	22.30	2.20	1.82
30A	25.60	2.10	23.70	2.10	1.92	25.50	2.20	2.23	21.50	2.10	1.75
72A	25.40	2.10	23.60	2.10	1.91	23.00	2.10	1.96	22.00	2.20	1.79
75A	28.00	2.30	23.90	2.10	1.94	23.20	2.10	1.98	22.40	2.20	1.82
97A	28.20	2.30	24.10	2.10	1.97	23.80	2.20	2.04	23.00	2.30	1.87
5A	28.30	2.40	24.40	2.20	2.00	24.00	2.20	2.06	23.60	2.40	1.92
9A	27.80	2.30	24.60	2.20	2.02	24.30	2.30	2.10	23.60	2.40	1.92
41A	34.10	2.80	24.80	2.20	2.04	25.50	2.40	2.23	27.50	2.70	2.24
54A	30.40	2.50	24.60	2.20	2.02	23.70	2.20	2.03	23.40	2.30	1.91
80A	31.30	2.60	24.60	2.20	2.02	25.40	2.40	2.21	25.80	2.60	2.10
91A	35.30	2.90	25.00	2.20	2.06	24.60	2.30	2.13	27.70	2.80	2.26
95A	31.40	2.60	25.00	2.20	2.06	24.90	2.30	2.16	24.30	2.40	1.98
13A	31.30	2.60	25.10	2.30	2.07	24.50	2.30	2.12	23.00	2.30	1.87
22A	41.10	3.40	25.20	2.30	2.09	26.40	2.50	2.32	23.90	2.40	1.95
45A	30.40	2.50	25.60	2.30	2.13	25.40	2.40	2.21	27.80	2.80	2.27
50A	37.80	3.10	25.40	2.30	2.11	25.40	2.40	2.21	31.00	3.10	2.53
82A	34.50	2.90	25.10	2.30	2.07	25.00	2.40	2.17	26.60	2.70	2.17
84A	34.30	2.90	25.10	2.30	2.07	25.80	2.40	2.26	28.40	2.80	2.32
90A	38.80	3.20	25.50	2.30	2.12	25.70	2.40	2.25	29.70	3.00	2.42
94A	32.10	2.70	25.90	2.30	2.16	25.80	2.40	2.26	26.50	2.60	2.16
2A	33.40	2.80	26.60	2.40	2.24	27.20	2.60	2.41	30.30	3.00	2.47
19A	32.90	2.70	26.20	2.40	2.20	25.80	2.40	2.26	25.20	2.50	2.05
23A	30.90	2.60	26.60	2.40	2.24	26.70	2.60	2.36	26.40	2.60	2.15
33A	30.00	2.50	26.10	2.40	2.18	25.40	2.40	2.21	24.20	2.40	1.97
37A	31.60	2.60	26.20	2.40	2.20	25.80	2.40	2.26	27.60	2.80	2.25
46A	34.20	2.80	26.20	2.40	2.20	25.70	2.40	2.25	25.60	2.60	2.09
52A	33.40	2.80	26.10	2.40	2.18	26.00	2.50	2.28	29.50	2.90	2.41
55A	33.60	2.80	26.40	2.40	2.22	26.00	2.50	2.28	28.80	2.90	2.35
87A	36.40	3.00	26.70	2.40	2.25	27.40	2.60	2.43	27.90	2.80	2.28
98A	33.80	2.80	26.50	2.40	2.23	26.70	2.60	2.36	30.20	3.00	2.47
15A	37.40	3.10	27.60	2.50	2.35	27.00	2.60	2.39	29.70	3.00	2.42
53A	39.10	3.30	27.50	2.50	2.34	28.00	2.70	2.50	32.20	3.20	2.63
64A	39.50	3.30	27.20	2.50	2.31	26.90	2.60	2.38	29.10	2.90	2.38
48A	32.90	2.70	28.20	2.60	2.42	27.80	2.70	2.48	28.60	2.80	2.33
58A	45.40	3.80	27.70	2.60	2.36	29.50	2.90	2.67	37.40	3.70	3.06
66A	37.60	3.10	28.30	2.60	2.43	29.20	2.90	2.64	34.10	3.40	2.79
79A	41.20	3.40	28.40	2.60	2.44	28.20	2.80	2.52	31.70	3.10	2.59
96A	37.20	3.10	28.20	2.60	2.42	28.30	2.80	2.53	31.00	3.10	2.53
99A	43.20	3.60	27.70	2.60	2.36	28.10	2.70	2.51	34.80	3.40	2.84
11A	37.90	3.20	29.10	2.70	2.52	28.40	2.80	2.55	32.30	3.20	2.64
21A	29.90	2.50	28.60	2.70	2.47	28.70	2.80	2.58	30.10	3.00	2.46
31A	35.40	3.00	28.60	2.70	2.47	28.70	2.80	2.58	28.90	2.90	2.36
43A	41.10	3.40	28.80	2.70	2.49	28.40	2.80	2.55	36.80	3.60	3.01
63A	43.00	3.60	29.30	2.70	2.55	32.10	3.20	2.97	42.00	4.10	3.44
83A	38.20	3.20	28.60	2.70	2.47	28.90	2.80	2.60	32.00	3.20	2.61
86A	32.10	2.70	42.90	2.70	4.21	27.90	2.70	2.49	28.40	2.80	2.32
93A	40.40	3.40	28.70	2.70	2.48	29.60	2.90	2.68	35.00	3.40	2.86
3A	42.70	3.60	30.10	2.80	2.64	30.00	3.00	2.73	35.00	3.50	2.86
38A	36.30	3.00	30.00	2.80	2.63	29.60	2.90	2.68	30.20	3.00	2.47
62A	44.40	3.70	29.90	2.80	2.61	28.80	2.80	2.59	32.50	3.20	2.66
81A	44.60	3.70	29.80	2.80	2.60	30.60	3.00	2.79	34.60	3.40	2.83
92A	42.50	3.50	29.40	2.80	2.56	29.60	2.90	2.68	34.10	3.40	2.79
4A	43.20	3.60	30.20	2.90	2.65	30.70	3.10	2.81	35.10	3.50	2.87
71A	53.20	4.40	30.20	2.90	2.65	30.80	3.10	2.82	42.30	4.10	3.46
74A	48.00	4.00	30.80	2.90	2.72	30.90	3.10	2.83	38.30	3.80	3.13
88A	38.60	3.20	30.50	2.90	2.68	30.00	3.00	2.73	32.00	3.20	2.61
59A	49.40	4.10	31.40	3.00	2.79	32.10	3.20	2.97	37.40	3.70	3.06
28A	36.70	3.10	32.00	3.10	2.86	31.60	3.20	2.91	29.70	2.90	2.42
29A	44.20	3.70	32.10	3.10	2.87	31.90	3.20	2.94	37.70	3.70	3.08
69A	44.00	3.70	31.90	3.10	2.85	31.50	3.20	2.90	42.20	4.10	3.45
16A	61.00	5.10	33.20	3.20	3.00	33.70	3.40	3.15	45.00	4.40	3.69
67A	50.00	4.20	34.10	3.30	3.11	32.50	3.30	3.01	37.40	3.70	3.06
85A	51.80	4.30	33.80	3.30	3.07	34.80	3.60	3.28	48.00	4.60	3.93
100A	53.30	4.40	34.60	3.40	3.17	36.90	3.90	3.53	53.00	5.10	4.35
8A	57.40	4.80	35.70	3.50	3.31	34.40	3.50	3.23	42.00	4.10	3.44
40A	55.60	4.60	35.50	3.50	3.28	35.70	3.70	3.39	50.00	4.80	4.10
61A	53.00	4.40	35.60	3.50	3.29	36.30	3.80	3.46	41.90	4.10	3.43
68A	56.00	4.70	35.70	3.50	3.31	35.00	3.60	3.30	47.10	4.60	3.86
70A	45.70	3.80	35.00	3.50	3.22	34.00	3.50	3.19	43.00	4.20	3.52
76A	59.40	5.00	35.60	3.50	3.29	36.90	3.90	3.53	50.20	4.80	4.12
56A	65.40	5.50	37.10	3.70	3.48	37.70	4.00	3.62	45.90	4.40	3.76
65A	55.60	4.60	38.30	3.90	3.63	37.60	4.00	3.61	45.90	4.50	3.76
78A	61.20	5.10	38.10	3.90	3.60	39.40	4.20	3.83	57.10	5.40	4.69
57A	65.40	5.40	39.10	4.00	3.73	37.90	4.00	3.65	48.90	4.70	4.01
60A	59.40	5.00	39.80	4.10	3.82	40.10	4.30	3.91	49.80	4.80	4.08
89A	64.20	5.30	46.00	4.90	4.62	44.00	4.90	4.40	61.40	5.80	5.04

**Table 2:** Frequency distribution of INR values before and after PT/INR line correction.

**Before PT/INR correction**						Total
	INR<2	2≤INR<3	3≤INR<4	4≤INR<5	INR≥5	
CoaguChek XS	31	42	29	11	7	120
UTMB Stago	42	58	17	3	0	120
ARUP Stago	38	55	21	6	0	120
BCS	32	42	27	15	3	119
Total	143	197	94	35	10	479
**After PT/INR line correction**						
CoaguChek XS (uncorrected)	31	42	29	11	7	120
UTMB Stago	43	54	17	5	1	120
ARUP Stago	40	48	22	9	1	120
BCS	39	37	24	8	11	119
Total	153	181	92	33	20	479

One-way repeated measures ANOVA was performed to investigate correspondence between methods both before and after PT/INR line correction. Because the raw and PT/INR line corrected data failed the Mauchly's Test for Sphericity (*p*=6.17e-59 before correction and *p*=1.30e-37 after correction), a Greenhouse-Geisser correction was applied to all ANOVA models. The p-values for one-way repeated measures ANOVA of the raw and corrected INR data for all methods were 3.52e-24 and 8.91e-14, respectively, indicating improved but statistically significant inter-method variation. In order to identify the source of the variation, paired t-test were performed both before and after PT/INR line correction, as shown in Table 3. While the p-values show that inter-method agreement improved for all methods following PT/INR correction, only the human thromboplastin based methods (BCS and uncorrected CoaguChek XS) showed agreement exceeding the *p*=0.05 threshold.

**Table 3:** Paired t-test analysis of INR results before and after PT/INR line correction.

**Before PT/INR line correction**			
	ARUP Stago	BCS	UTMB Stago
BCS	<2e-16	–	–
UTMB Stago	<2e-16	<2e-16	–
CoaguChek XS	1e-15	0.08	<2e-16

**After PT/INR line correction**			
BCS	2.6e-07	–	–
UTMB Stago	9.5e-09	1.7e-09	–
CoaguChek XS	9.2e-12	0.47	8.0e-14

## 2 Experimental design, materials and methods

The study protocol was approved for verbal informed assent by the Institutional Review Board of the University of Texas Medical Branch (UTMB) and Roche Diagnostics USA (Indianapolis, IN). Warfarin therapy patients were recruited from March to May 2015 during routine visits to monitor their PT/INR at UTMB's anti-coagulation clinic and all POCT measurements were performed on the CoaguChek XS by a single trained nurse. The warfarin patients seen in the anti-coagulation clinic were routinely monitored and Coumadin dosage changed using the CoaguChek XS results. POCT PT/INR results >5.0 were sent to the main laboratory for confirmation. The POCT PT/INR was determined prior to asking the patient if they would be willing to be included in the study. If willing to participate the subject was taken to phlebotomy to have a venous blood sample drawn in 3.2% citrate, where the first 1 ml of venous blood was discarded. The sample was then transferred to the laboratory where it was centrifuged at >3000x*g* for 10 min to prepare platelet poor plasma. Within 4 h of sample collection an aliquot of the platelet poor plasma was analyzed for PT/INR in the main UTMB Pathology laboratory on the Stago Star Evolution (Diagnostic Stago Inc., Mount Olive, NJ). Additional aliquots were prepared and frozen for later analysis of PT INR on the Stago Star Evolution (Stago, Parsippany, NJ) located at ARUP Labs (Salt Lake, UT) and the Siemens BCS (Siemens Medical Solutions USA, Inc., Malvern, PA) located at CPL (Austin, TX). The Stago Star Evolution used rabbit brain thromboplastin (UTMB ISI=1.25; ARUP ISI=1.28) and the CoaguChek XS and Siemens BCS used recombinant human thromboplastin (ISI=1.01 and 1.02, respectively).

The 20 normal (non-warfarin) samples and 100 warfarin treatment samples were included in this investigation. All warfarin therapy samples were obtained from patients who have been on stabilized warfarin therapy for at least one month to allow both short half-life (Factor VII) and long half-life vitamin K-dependent clotting factors to attain therapeutic equilibria. Patients included in this study are expected, based on their medical history, to be negative for anti-phospholipid antibodies, have hematocrit values in the 25–55% range, and have taken no additional anticlotting medication such as aspirin.

### 2.1 PT INR measurement

PT INR measurements were performed using the POCT CoaguChek XS system (UTMB anticoagulation clinic), core laboratory Star Evolution (at UTMB and ARUP), and BCS XP (at CPL). INR measurements were obtained for all 120 samples, using all methods except for one BCS sample. The CoaguChek XS system detects coagulation in a drop of whole blood while the central lab methods employ citrated plasma (as described above). The CoaguChek XS and the BCS XP use a human recombinant thromboplastin while the Star Evolution uses rabbit brain thromboplastin (STA Neoplastin). A single CoaguChek XS device and only one lot of test strips were used for this study.

### 2.2 Calibrant standards and controls

Poller calibrants (European Concerted Action on Anticoagulation or ECAA PT/INR plasma set, batch 1591-6) were purchased from Hart Biologicals Ltd. (Hartlepool, England). The frozen standards and controls were stored and prepared according to vendor specifications prior to analysis in duplicate on the three central laboratory analyzers.

### 2.3 PT/INR line correction

INR data from the Stago and BCS XP were corrected using the regression equation generated from ln(PT observed) vs ln(INR certified) calibration plots obtained from central lab analysis of the ECAA Poller calibrant [2], [3], [4]. The certified Poller INR values were obtained from the manufacturer and the values for the specified thromboplastins were employed (See Table 2A, Ref. [1]). Linear regression analysis was performed to generate the PT/INR regression line (*y*=*mx*+*b*), where *x*=ln(INR certified), *y*=ln(PT observed), *m*=slope, *b*=intercept, and *y*=*mx*+*b*. The PT/INR line corrected sample INR values (shown in Table 1) were generated using the rearranged equation *x*=(−*b*/*m*)+(1/*m*)*y* by inputting *y*=ln(sample PT) and raising *x*=ln(INR corrected) to the natural exponent (e^ln(INR corrected)^=INR corrected).

### 2.4 Statistical Analysis

Pearson's chi-square testing, paired t-tests, and one-way repeated measures ANOVA were performed using R 3.4.
